# Antizyme inhibitor family: biological and translational research implications

**DOI:** 10.1186/s12964-023-01445-1

**Published:** 2024-01-02

**Authors:** Qiaohui Feng, Huijie Wang, Youcheng Shao, Xiaoyan Xu

**Affiliations:** 1 Department of Breast Surgery, The First Hospital of China Medical University, Shenyang, Liaoning 110001 PR China; 2 Department of Pathophysiology, College of Basic Medical Science, China Medical University, No. 77 Puhe Road, Shenyang North New Area, Shenyang, 110122 Liaoning Province PR China

**Keywords:** Antizyme inhibitors (AZINs), Polyamine, RNA editing, Microenvironment, Therapy

## Abstract

Metabolism of polyamines is of critical importance to physiological processes. Ornithine decarboxylase (ODC) antizyme inhibitors (AZINs) are capable of interacting with antizymes (AZs), thereby releasing ODC from ODC-AZs complex, and promote polyamine biosynthesis. AZINs regulate reproduction, embryonic development, fibrogenesis and tumorigenesis through polyamine and other signaling pathways. Dysregulation of AZINs has involved in multiple human diseases, especially malignant tumors. Adenosine-to-inosine (A-to-I) RNA editing is the most common type of post-transcriptional nucleotide modification in humans. Additionally, the high frequencies of RNA-edited AZIN1 in human cancers correlates with increase of cancer cell proliferation, enhancement of cancer cell stemness, and promotion of tumor angiogenesis. In this review, we summarize the current knowledge on the various contribution of AZINs related with potential cancer promotion, cancer stemness, microenvironment and RNA modification, especially underlying molecular mechanisms, and furthermore explored its promising implication for cancer diagnosis and treatment.

## Introduction

**Fig. 1:**
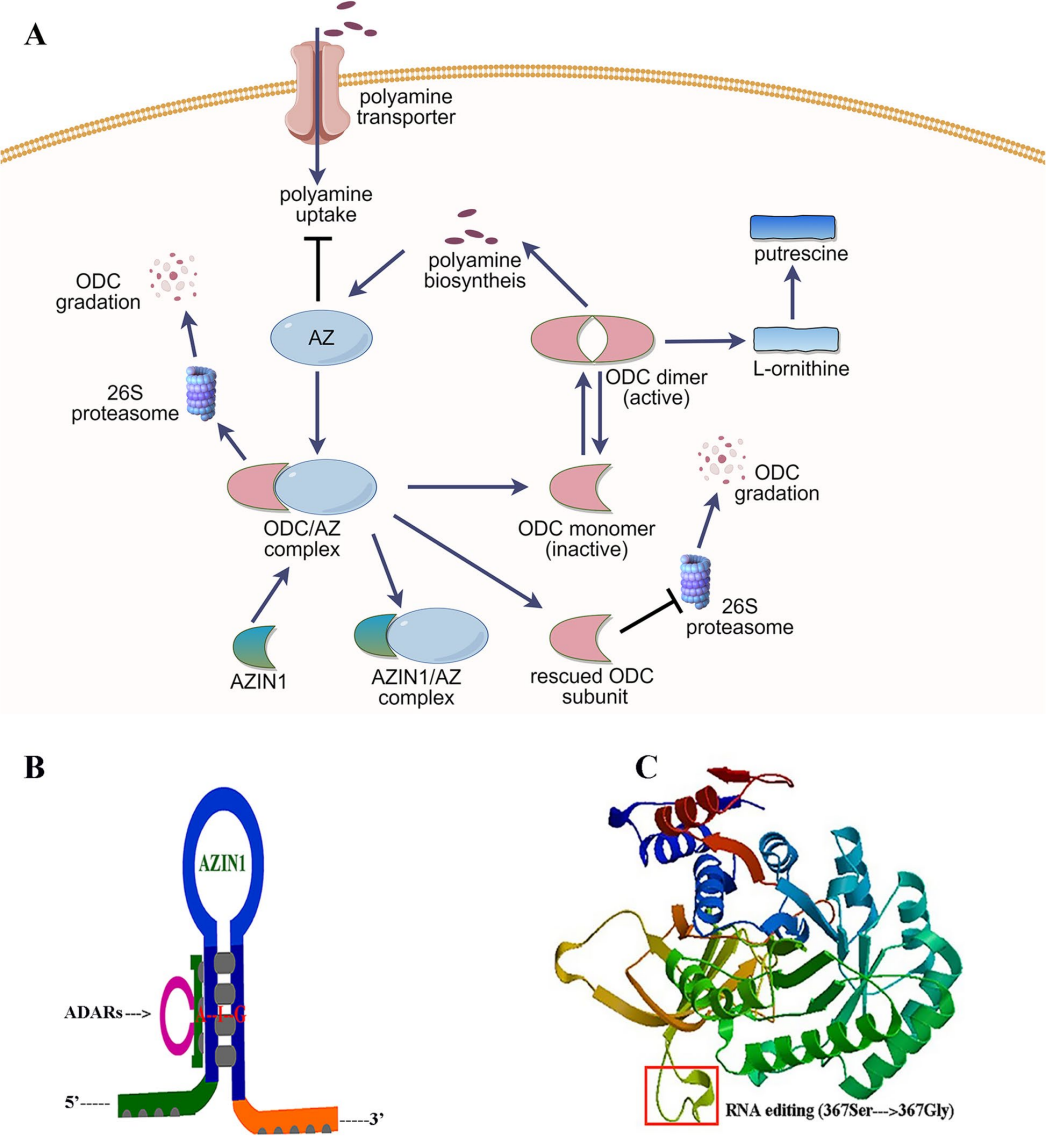
Schematic diagram of AZIN1. **A** Schematic diagram of AZIN1-mediated polyamine metabolic pathway. The figure was drawn by Figdraw. **B** Pattern diagram of AZIN1 edited by ADAR1. **C** The alteration of 3D structure on serine-to-glycine substitution at residue 367 of AZIN1 edited by ADAR1

Metabolism of polyamines is of critical importance to physiological processes, such as cell growth, proliferation, differentiation, and apoptosis [1–3]. Abnormal regulation of polyamine metabolism appears to be captured in various human diseases [4, 5], notably malignant neoplasms [6]. To maintain continual proliferation, cancer cells require sustained and elevated intracellular polyamine pools, which is directly linked to oncogenes, including *MYC*, *JUN*, *FOS*, *KRAS* and *BRAF* [5, 7]. Furthermore, emerging data showing that polyamines also have anti-inflammatory and immunosuppressive properties in the tumor microenvironment (TME) [8, 9]. Most notably, the two rate-limiting enzymes of polyamine biosynthesis are ornithine decarboxylase (ODC) and S-adenosylmethionine decarboxylase (AMD1), which are both direct transcriptional targets of MYC [10, 11]. Generally speaking, the organized regulation of ODC, greatly depends on the balance between agonists and antagonists in the physiological state [12]. As naturally occurring antagonists, antizyme family (AZs), composed of three kinds of small protein-AZ1, AZ2 and AZ3, have involved in regulating polyamine biosynthesis by means of directly binding to ODC, thereby accelerating the degradation of ODC via the 26S proteasome, and subsequently inhibiting extracellular polyamine uptakes [13–15] (Fig. 1A). In human, AZ1 and AZ2 are ubiquitously expressed, while AZ3 can only be tested in testis [16]. Furthermore, the activity of AZs is also tightly controlled by AZINs through assembling and constructing AZs-AZINs complex [17]. There are two naturally occurring antizyme inhibitors, including AZIN1 and AZIN2, homologue proteins of ODC. AZINs have access to binding to AZs directly, and thereby stabilize ODC homodimer and negate the effect of AZs-ODC on polyamine metabolism. Notably, compared with ODC, AZINs directly combine to AZs much more tightly resulting in antizyme segregation [18, 19]. Consequently, AZINs act the central part in modulating the function of ODC catalytic activity [20–22]. Although aberrant regulation of AZINs frequently occurs in human diseases, the underlying mechanisms have not been clarified clearly and comprehensively.

Recently, RNA editing, an emerging study of epigenetic machinery, has started to be gradually thrown light upon. RNA editing is a post-transcriptional process in which nucleotide changes can be detected directly by RNA sequencing, which involved in crucial physiological and pathological processes [23]. Desregulation RNA editing was widely indicated in many human malignant diseases, especially cancers [24, 25]. In mammal, RNA editing mainly consists of adenine (A) to inosine (I) and cytosine (C) to uracil (U), which are specifically catalyzed by adenosine and cytidine deaminases, respectively [26, 27]. Notably, the most frequent type of RNA editing event is conversion of A to I, subsequently recognizing the inosine (I) as a guanosine (G) in homo sapiens [28, 29]. A-to-I RNA editing is mediated by the double-stranded RNA (dsRNA) specific adenosine deaminase acting on RNA (ADARs) family, including ADAR1, ADAR2 and ADAR3 [30–32] (Fig. 1B). In view of above facts, RNA editing represents a novel mechanism to control organism homeostasis independent of somatic DNA mutation. Transcriptome sequencing revealed that A-to-I RNA editing of AZIN1 accounts for high modification rate in various cancers [33–35], which is specifically regulated by ADAR1 [36]. Recent evidence has strengthened this signature by correlating highly frequent AZIN1 edited site with various cancers occur and pathogenesis to regulate malignant phenotype transformation of cells [33–35, 37]. Therefore, it is rather reasonable to reckon that interpretation of clinical value and prognostic indication of AZIN1 from the perspective of A-to-I RNA editing.

Here, we comprehensively reviewed the various roles of AZINs and summarized underlying polyamine-dependent and independent molecular mechanisms in human diseases. In a word, we earnestly hope that our research can construct a solid bridge from basic research to clinical practice.

## Structure characteristics and expression atlas of AZINs across human diseases

### The gene structural landscape of AZINs family

**Fig. 2:**
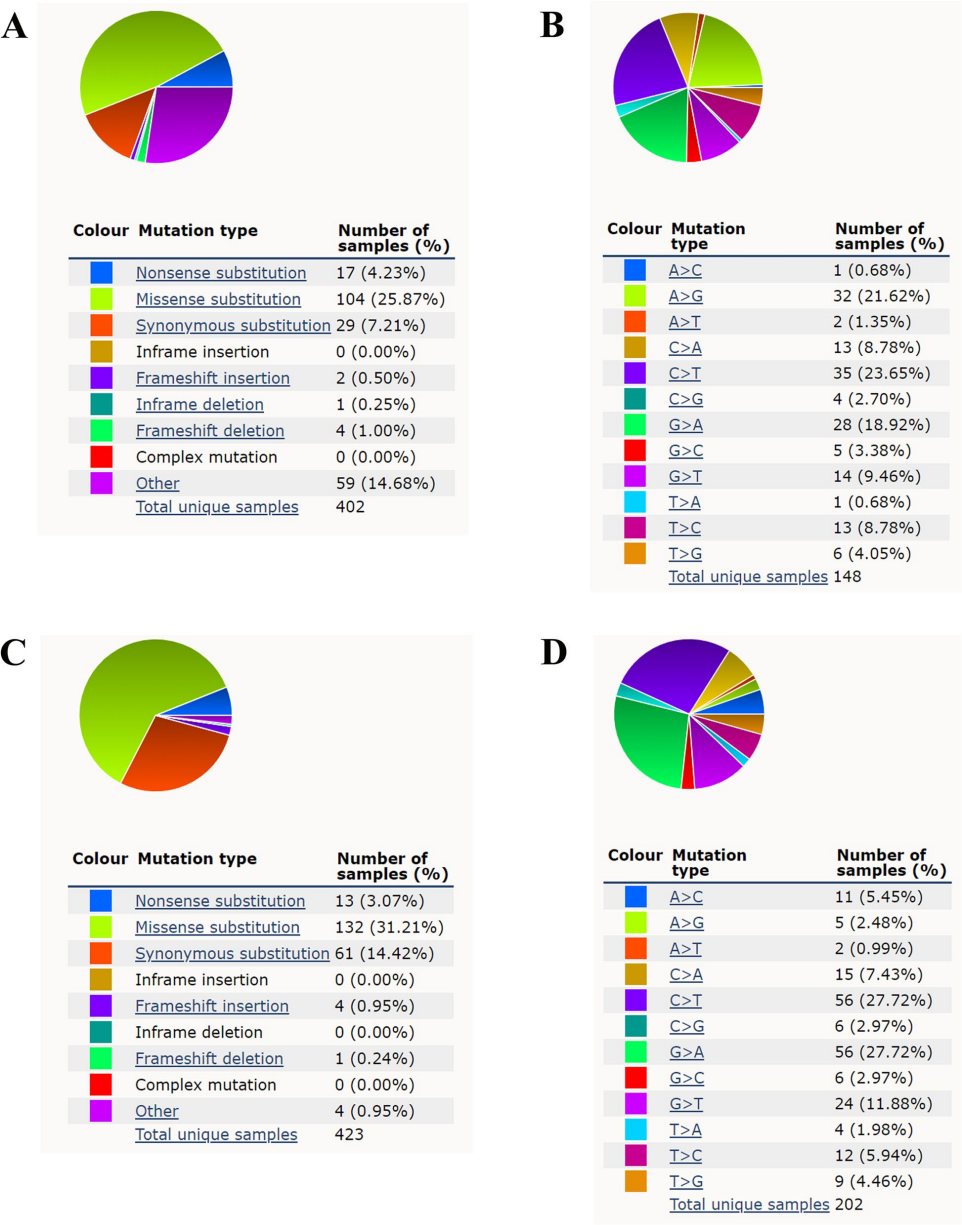
The mutation distribution of AZIN1 and AZIN2 in cancers. **A** and **B** Pie-chart showing the distribution and percentage of the mutation type of AZIN1 in cancers according to COSMIC database. **C** and **D** Pie-chart showing the distribution and percentage of the mutation type of AZIN2 in cancers according to COSMIC database. The table shows the number of samples recorded as having a particular type of mutation, with the number in brackets giving the percentage of samples with that type of mutation

The human AZIN1 gene is mapped to chromosome 8q22.3 in reverse chain, and stretches from 103,838,536 to 103,876,905, including 13 exons and 12 introns (RefSeq-GRCh37.p13) [38]. Due to high throughout sequencing and automatically annotated method, AZIN1 mutation event was comprehensively characterized. The most common AZIN1 mutations across cancers are missense substitutions. Other mutations include synonymous substitutions, nonsense substitutions, and frameshift deletions (Fig. 2A). The most common substitution mutations are C > T, G > A, and A > G (COSMIC) (Fig. 2B). Although the clinical prognosis of most AZIN1 mutation sites remains unknown, it was shown that a single -nucleotide polymorphism (SNP) variant rs2679757 was related to the risk of liver fibrosis in Chinese patients with HBV-associated chronic liver diseases [39]. Interestingly, although AZIN1 possesses 13 exons, only 10 of all exons are involved in encoding the protein. There is no wonder that alternative splicing event of AZIN1 was tightly controlled by multiple regulatory systems under physiological conditions. For instance, Paris et.al reported that an intronic SNP produces the new alternative splice site and affects post-transcriptional regulation of AZIN1, thus conferring diversity of expression and function [40]. Besides, a minor allelic SNP variant (SNP C/A 34689) in the 12th exon of AZIN1 favors the expression of AZIN1 splice variant SV2 that can slow down the fibrosis rate by inhibiting the expression of fibrogenic genes [40].

Both of AZIN1 and AZIN2 have similar structure character. The human AZIN2 (originally called ODC-p) gene spans about 81 kb pairs and is located on chromosome 1p35.1, containing 11 exons and 10 introns of variable length in humans [41]. The first two exons of AZIN2 are noncoding, and the ORF starts at exon 3 and ends at exon 11, coding the protein of 460 amino acid. The distribution of different mutation types for AZIN2 is shown (Fig. 2C, D), and eight alternative spliced forms of AZIN2 have been detected. Nevertheless, there is no additional study to be reported on the expression of isoforms of human AZIN2 and the potential biological function of the spliced forms.

### The protein of AZIN1 and AZIN2

**Fig. 3:**
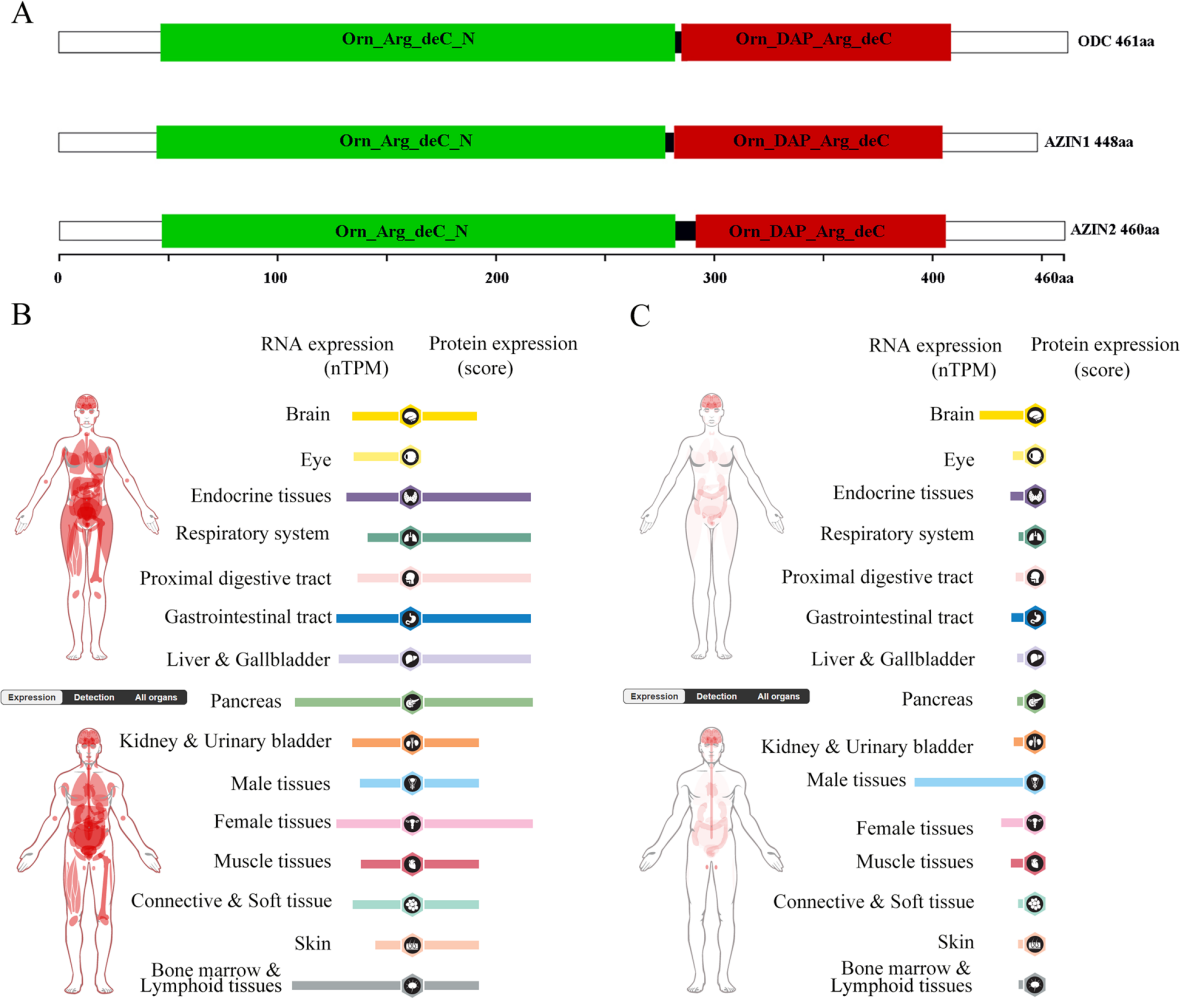
The expression of AZIN1 and AZIN2 in human cancers. **A** Ideograph of AZIN1, AZIN2 and ODC proteins for showing domains. **B** Expression profile for AZIN1 in human cancers. **C** Expression profile for AZIN2 in human cancers according to The Human Protein Atlas. Left stands for mRNA expression and right represent protein expression

The widely recognized protein encoded by the AZIN1 gene possesses 448 amino acid residues, which are comprised of two functional domains, including the Orn_Arg_deC_N, pyridoxal-dependent decarboxylase, as the pyridoxal binding domain, and Orn_DAP_Arg_deC, pyridoxal-dependent decarboxylase, as the C-terminal sheet domain (Evidence from EBI-pfam) (Fig. 3A). A previous study reported that mutating several residues within the AZ binding region (117–140 amino acids) of AZIN1 failed to bind to AZ, indicating that this region is indispensable to the interaction of AZIN1 and AZ [42]. Although the AZIN1 protein contains amino acid residues that constitute the active sites of ODC, AZIN1 has no homology with the peptide sequence of mammalian ODC carboxyl terminus that contributes to the rapid turnover of ODC. It has previously been reported that cellular localization of AZIN1 varies during the cell cycle, from cytoplasmic localization during interphase to centrosomic localization during mitosis, thereby indicating a role for AZIN1 in the mitotic process [43]. Much more importantly, the alterated AZIN1 balance leads to numerical centrosomal defects, which suggests a role for ubiquitin-independent proteasome degradation during centrosome duplication [44]. These findings have likely revealed AZIN1 location play a crucial role in determining various function for organism. Reported post-translational modifications for AZIN1 has been ubiquitination recently, which is associated with its degradation. In terms of degradation manner, AZIN1 is much different from ODC. Moreover, AZs directly binding to AZIN1 induces conformational change of AZIN1, which may hide the degradation signal of AZIN1 to inhibit its ubiquitination [19]. Thus, the above findings have revealed the location and cleavage manner of AZIN1, which determines the crucial function for organism.

It was puzzled to all that AZIN2 gene encodes the protein of 460 amino acids in the light of over twofold gene span than that of AZIN1. Additional four isoforms are produced by alternative splicing and encode proteins of 480, 374, 362, and 204 amino acids, respectively. Interestingly, AZIN2 was initially reported to have ornithine decarboxylase activity [45] and arginine decarboxylase activity [46], but later it was reported that it possessed neither of them [47–49]. Likely AZIN1, AZIN2 comprise two domains, including the Orn_Arg_deC_N and Orn_DAP_Arg_deC (Evidence from EBI-pfam) (Fig. 3A). The similarity between human AZIN1 and AZIN2 is 64% in protein sequences. Besides, the coding sequences between human AZIN1 and AZIN2 present the highest divergences in the N- and C-terminal regions, in contrast to the best conservation (86% similarity) found in the putative antizyme binding element (AZBE) (Evidence from NCBI-Blast). Regarding its physiological role, spatial and temporal analyses of AZIN2 expression in the mouse testis suggested that AZIN2 might have a role in spermiogenesis. AZIN2 represents a parallel group independent of AZIN1, and AZIN2 sequence is much more similar to ODC than its counterpart AZIN1 [50]. But the sub-cellular location of AZIN2 is different from AZIN1, and AZIN2 is located in the endoplasmic reticulum–Golgi intermediate compartment and cis-Golgi network [41]. Moreover, inhibitors of the lysosomal function partially prevented the effect of the proteasome inhibitor MG132 on AZIN2, which suggests that the degradation of AZIN2 is mediated by an alternative route to that of proteasome [49]. Based on the above findings, the highly selective localization of AZIN2 and its high similarity to ODC may suggest itself more tissue-specific function.

## The landscape of AZINs expression in human malignant diseases

The expression of AZINs is tightly regulated during embryogenesis and development, both spatially and temporally. However, the dysregulation of AZINs is frequently reported in various human diseases.

The AZIN1 is expressed in human all tissues, and its RNA transcription level is significantly higher in the human brain tissue compared to other tissues (Fig. 3B). The highest mRNA level is shown in the pancreas and adrenal glands of murine [51]. These differences suggest that AZIN1 may regulate various biological processes in species specificity and tissue specificity manner. Interestingly, a growing number of studies reported that AZIN1 expression increased in most cancer types [52], including gastric cancer [53], ovarian cancer [54] and prostate cancer [55] and colorectal cancer [56]. To be note, AZIN1 was down-regulated in the transcription panel of hypoxia related lung cancer biomarker [57], but lacking direct experimental evidence to support its possible regulation with hypoxia during lung cancer. These reported evidences suggest necessity of further elucidation about the role of AZIN1 in human diseases.

Previous research depicted that AZIN2 RNA expression appears to be restricted to brain and testis [58] (Fig. 3C). And recent studies showed that the expression of AZIN2 was upregulated in the nervous system, megakaryocytes, type 2 pneumocytes of the lung, and gastric parietal cells, and co-localized with H–K-ATPase beta subunit. In addition, AZIN2 mRNA was more abundant than AZIN1 mRNA in testis, epididymis, adrenal gland, brain and lung [59]. AZIN2 is highly expressed in various cells with secretory or vesicle transport activity showing that AZIN2 is significantly involved in transmembrane transport by regulating polyamine metabolism [60–62]. Hypoxia-induced AZIN2 high expression may contribute to cisplatin resistance by promoting the epithelial-mesenchymal transition (EMT) in non-small cell lung cancer (NSCLC) [63].

## AZINs act as the regulators in human disease through a polyamine-dependent pathway

The polyamines including putrescine, spermidine, and spermine are small cations which can bind to DNA and RNA, found in all cells, and essential for cell growth [64]. A few proteins have been reported as polyamine transporters to regulate polyamine uptake [65]. For example, ATP13A2 has been shown to be a lysosomal polyamine exporter [66], and SLC12A8 was originally regarded as another polyamine importer [67], but now is designated as an amino-acid and a nicotinamide mononucleotide transporter [68]. Recently, ATP13A3 has been termed as a major mammalian polyamine transporter [69].

ODC initials the first rate-limiting step in polyamine biosynthesis, triggering the change from L-ornithine to putrescine. AZINs emancipate ODC from ODC-AZs complex, and prompt polyamine synthesis and extracellular polyamine uptake. There is no doubt that polyamine signaling pathway is a convenient expressway for AZINs to perform functions. Tumor cells overexpressing AZIN1 exhibit higher sensitivity to polyamine analogs due to osmotic imbalance caused by the elevated cellular polyamine level [70]. It was reported that AZIN1 overexpression increases the level of spermidine and spermine, along with a modest increase in putrescine thereby promoting cell proliferation, enables cells to grow in low serum and anchorage-independent environment [22].

Accordingly, overexpression of AZIN1 was accompanied by increase of ODC activity in breast cells. Furthermore, overexpressed-AZIN1 cells exhibited high proliferation. Interestingly, putrescine increased markedly, while spermine decreased in the cells of AZIN1 overexpression [20], and further experiments will be applied to clarify the mechanism. Accordingly, AZIN1 expression is high in the vaso-pressinergic and oxytocinergic neurons of the paraventricular and supraoptic nuclei, and is further increased in response to hypertonic stressors by modulating polyamine level [71].

In animal models, AZIN1-knockout mice exhibit alterations in expression of genes involved in polyamine biosynthesis, embryonic development, and cell proliferation, indicating the importance of AZIN1 in maintaining polyamine homeostasis, modulating cellular proliferation, and inducing transformation [72]. On one hand, AZIN1-deletion mice died at birth (P0) with abnormal liver morphology and significantly downregulated level of ODC, putrescine and spermidine [73]. On the other hand, transgenic mice with tissue-specific AZIN1 overexpression have increased tumor incidence, elevated level of polyamine, and triggered enhanced proliferation [52].

Like AZIN1, it has been reported that the overexpression of AZIN2 confers growth advantage to NIH3T3 cells, similar to AZIN1, probably by increasing polyamine level [74]. The role of AZIN2 is important in testicular cells by modulating polyamine concentration, testosterone synthesis and sperm function [75]. Recently, AZIN2 expression was also restricted to the adrenal medulla and the Langerhans islets in the adrenal glands and pancreas [61]. It is suggested that AZIN2 may have a role in the regulation of reproductive and secretory function. AZIN2 would affect cellular processes through its action on modulation of intracellular polyamine pools and target proteins in specific mammalian cells.

## The participation of AZINs in diseases related with other signaling pathways

AZIN1 is capable of regulating the renal and cardiac fibrosis through transforming growth factor-β (TGF-β) signaling pathway. Forced expression of miR-433 suppressed the expression of targetted gene AZIN1, while AZIN1 overexpression suppressed TGF-β signaling and the fibrosis response [76]. Furthermore, AZIN1 knockdown could promote proliferation and differentiation of cardiac fibroblasts into myofibroblasts accompanied with an activation of TGF-β/Smad3 signaling pathway [77]. But the direct relationship between AZIN1 and TGF-β and their functional roles in the regulation of renal and cardiac fibrosis needs to be further clarified.

AZIN1 enhanced migration of prostate cancer cells independent of polyamines, by modulating production of matrikines released from type IV collagen [78]. Much importantly, AZIN1 is involved in the regulation of centrosome duplication and cell cycle. Besides, AZIN1 neutralizes AZs-mediated degradation of oncoproteins, including cyclin D1, Aurora-A and DNp73 [44]. Recently, it is reported that RNA-edited AZIN1 has a stronger capacity of inhibiting AZs-mediated oncoproteins degradation than wild-typed AZIN1 [33].

### Cell cycle regulation of AZIN1

Cyclin D1 serves as an active regulator in cell cycle and acts as a transcriptional co‐regulator [79]. AZs binds cyclin D1 and directs it to degradation by the proteasome in the ubiquitination-independent pathway. The interaction of AZIN1 with AZs represses AZs-mediated degradation of cyclin D1 and allows normal cell cycle progression [80]. A previous study has confirmed that AZIN1 directly binds to cyclin D1 and restrains its degradation [80]. In addition, AZIN1 inhibits the degradation of DeltaNp73 functioning as an apoptosis suppressor when exposed to genotoxic stress [81]. AZIN1 knock-out mice showed upregulated level of Stag2 and Chek1, which are critical regulators in cell cycle [73].

### AZIN1 in centrosome modulation

Centrosome amplification occurs at early stage of tumorigenesis. Silencing of AZIN1 reduces numerical centrosome abnormalities, whereas overexpression of AZIN1 causes centrosome over‐duplication [17]. AZs mediates the degradation of Aurora-A in a proteasome-dependent but ubiquitination-independent pathway. Co-expression of Aurora-A and AZIN1 suppresses AZs function and abrogates AURKAIP1-mediated degradation of Aurora-A, leading to aneuploidy and centrosome abnormality [82]. It has been demonstrated that AZIN1 regulates tumor behavior by modulating centrosome duplication and upregulating loricrin to promote differentiation of cancer cells [83]. AZIN1 could inhibit Mps1 degradation, and cause centrosome amplification in asynchronous growth of HeLa cells [83]. As stated above, it is worthy to be further investigate whether AZIN1 involves in the regulation of these proteins by binding and inactivating AZs.

## The role of AZINs in cancer stemness and microenvironment

Limited nutrients and oxygen are often the feature of early avascular tumors and late-stage necrotic tissues, and high level of AZIN1 can confer growth advantage, thus aiding tumor formation [22]. The increase is most prominent in low serum conditions, suggesting that AZIN1 can overcome the limitation of growth factor. AZIN1 was highly edited in hematopoietic stem and progenitor cells (HSPCs). RNA-edited AZIN1 translocated to the nucleus, enhanced binding affinity for DEAD box polypeptide 1 to change the chromatin distribution of the latter, and altered expression of multiple hematopoietic regulators that ultimately augment HSPC differentiation [84]. Above results will facilitate further studies into RNA editing in hematopoietic cells under physiological and pathophysiological conditions.

Recent evidence has shown that the tumor microenvironment plays a crucial role in cancer progression. Cancer associated fibroblasts (CAFs) represent an abundant cell population in the tumor microenvironment [85], and promote several tumor cell characteristics including aggressiveness enhancement [86]. RNA-edited AZIN1 enhances colorectal cancer (CRC) stemness and appears to drive the metastatic processes. As colorectal cancer stem cells (CSC) markers, the level of OCT4 and SOX2 was substantially higher in spheroids overexpressing RNA-edited AZIN1 [87]. Hyper-editing of AZIN1 enhances the invasive potential of CAFs in colon [88]. However, how RNA-edited AZIN1 enhances CRC stemness and the RNA-edited level of AZIN1 in CAFs is unclear. The clinical significance of RNA editing in CAFs also remains unexplored. Recently, we have demonstrated that RNA-edited AZIN1 promotes tumor angiogenesis through delaying c-Myc degradation by OAZ2-mediated ubiquitin independent proteasome pathway to increase IL-8 [89], which suggests an important contribution of RNA-edited AZIN1 to the tumor vascular microenvironment and highlights its translational potential. Moreover, recent advances in immunotherapy have brought to light the importance of cancer research in the context of its microenvironments. Thus, it is necessary and urgent to enhance knowledge of the effects of wild-typed and RNA-edited AZIN1 on the function of various cell types in the TME, with particular emphasis on diverse immune cells.

## RNA modification of AZINs in human diseases

RNA modification occurs on all four nucleotides in nature: A, U, C, and G [90]. In RNA levels, there are more than 170 modifications, including N^1^-methyladenosine (m^1^A), N^6^-methyladenosine (m^6^A), 3-methylcytosine (m^3^C), 5-methylcytosine (m^5^C), 7-methylguanosine (m^7^G), N^1^-methylguanosine (m^1^G), and adenosine-to-inosine editing [91, 92]. RNA editing is a modified type at the RNA level, belonging to post-transcriptional modification without altering its template genomic DNA. The predominant form of RNA editing observed in humans is the conversion of adenosine(A) to inosine (I), whereby inosine is recognized as guanosine by the translational machinery. This conversion process is facilitated by adenosine deaminases that act on RNA (ADARs). Among the ADAR enzymes, namely ADAR1, ADAR2, and ADAR3, there is a high degree of conservation across metazoans [93]. While ADAR1 and ADAR2 exhibit some distinct target specificities, there is also overlap in their editing preferences. The precise regulatory mechanisms governing A-to-I editing at specific sites are not yet fully understood, although the accessibility of ADAR to target substrate RNAs and protein–protein interactions that directly modulate ADAR enzymatic activity have been suggested as significant factors. To date, a substantial number of high-confidence A-to-I RNA editing sites, numbering in the millions, have been identified in humans. The majority of these sites are found within primate-specific Alu sequences, exhibiting a relatively low level of editing.

AZIN1 pre-mRNA editing was widely indicated in human diseases, especially human cancers by Transcriptome sequencing directly [33, 34]. However, the events that AZIN2 was edited or unedited remain obscure for long due to various reasons, including shorter half-life than AZIN1. Therefore, we mainly focused on the RNA editing of AZIN1 in the followings. RNA editing event serves as a landmark research in AZINs. RNA editing S367G in AZIN1 is a high-frequent molecular event by parallel DNA capturing and sequencing [94]. AZIN1 editing greatly depends on AZIN1 pre-mRNA secondary structure and the potential complementary sequence [31], which is specifically regulated by ADAR1 [36]. A-to-I RNA editing of AZIN1 protein-coding regions is highly selective and conserved, resulting in deamination of specific adenosine residues. The specific edited substitution characterized by serine (S) to glycine (G) at residue 367, located in β-strand 15 (β15), leading to a conformational change, promoting cytoplasmic-to-nuclear translocation, confers tumor more malignant and aggressive potential [33, 95] (Fig. 1C). The edited residue is located in the C-terminal sheet domain responsible for binding with AZ, and the resultant conformational change leads to a stronger binding affinity of AZIN1 to AZ. Compared with wild type AZIN1, edited-AZIN1 obtains a stronger binding ability with AZs, promoting protein stability and tumor cell proliferation through inhibiting AZs-mediated degradation of ODC and cyclin D1 [33]. In contrast, recent study refers to another possibility, reporting that nuclear translocation of RNA-edited AZIN1 is associated with certain direct binding proteins like myosin-9, alpha-smooth muscle actin (ACTA2) and actin gamma 1 (ACTG1) [95]. In addition, we also demonstrated that RNA editing sites are found mostly in non-coding regions [25]. Noncoding editing sites are highly specific, and are characterized with perfect duplex formed by Alu repetitive sequences. Furthermore, the underlying molecular mechanisms of RNA editing might be entirely unexpected and may have evolved from unexpected molecular processes. More RNA editing sites of AZIN1 in non-coding region are available at RADAR (Rigorously Annotated Database of A-to-I RNA Editing, http://rnaedit.com/).

**Fig. 4:**
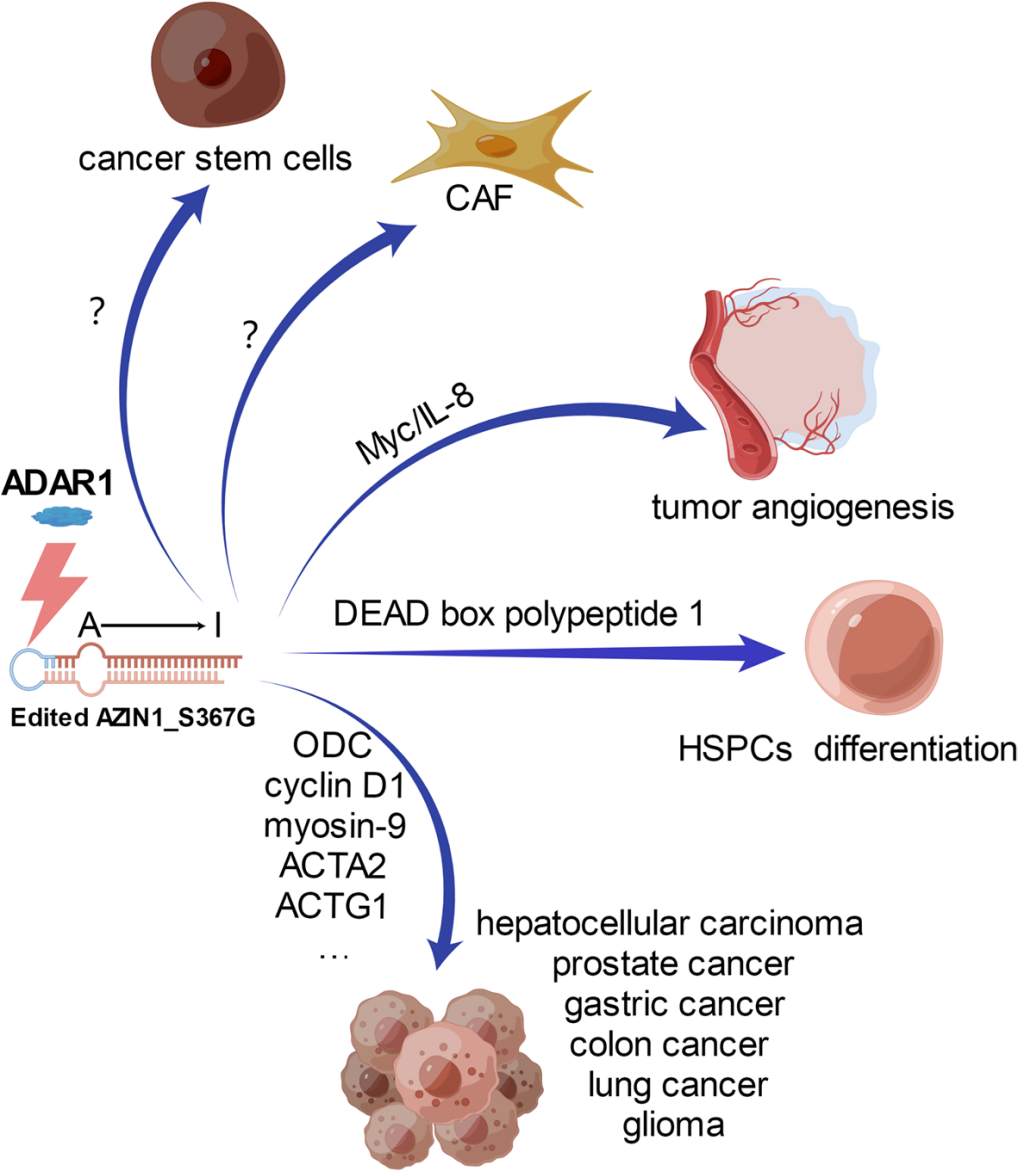
Schematic summary of the role of RNA-edited AZIN1 in tumor. RNA-edited AZIN1 enhances CRC stemness and appears to drive the metastatic processes. Hyper-editing of AZIN1 enhances the invasive potential of CAFs in colon. How RNA-edited AZIN1 enhances CRC stemness and regulation of CAFs is unclear. RNA-edited AZIN1 promotes tumor angiogenesis through delaying c-Myc degradation by OAZ2-mediated ubiquitin independent proteasome pathway to increase IL-8, enhances binding affinity for DEAD box polypeptide 1 to promote HSPC differentiation, and promotes protein stability and tumor cell proliferation through inhibiting degradation of ODC and cyclin D1. RNA-edited AZIN1 is associated with certain direct binding proteins like myosin-9, ACTA2 and ACTG1. The figure was drawn by Figdraw

Recent studies put forward an alternative possibility, revealing that A-to-I RNA editing of AZIN1 may also be a pivotal modulator for cell proliferation and oncogenesis (Fig. 4). The modification of AZIN1 RNA level may be another mechanism to involve in multiple physiological and pathological processes. Recent evidence from clinicopathological analyses has strengthened the signature by correlating elevated RNA-editing level of AZIN1 with the risk of liver cirrhosis, tumor recurrence, and worse prognosis [33]. Then it also was reported that RNA-editing S367G in AZIN1 is highly frequent in malignant glioma cells U87MG [96], and non-small-cell lung cancer samples and cell lines [34]. Chen et al. reported that A-to-I RNA editing of AZIN1 is significantly increased in HCC specimens. Intriguingly, the editing level of AZIN1 gradually increased during HCC pathogenesis ranged from normal, adjacent tissues and clinically verified HCC. AZIN1 RNA-editing level in primary tumor tissues is significantly correlated with lymph node metastasis in gastric cancer patients [35]. Other studies demonstrated that edited AZIN1 confers the invasive potential of CAFs in colon cancer and predicts tumor invasiveness in colorectal cancer [88]. And RNA-edited AZIN1 leads to enhanced cellular aggressiveness, and is associated with worse prostate cancer outcomes [95]. Besides, in our research, edited AZIN1 acted as a “driver,” significantly increasing proliferation in both MCF10A and Ba/F3 cells [25]. Thus, A-to-I RNA editing of AZIN1 contributes to cancer initiation, progression, and therapeutic response. While unlike AZIN1, the RNA editing event on AZIN2 has not been identified so far.

While it is possible that many of above mentioned modifications interact, so far, it is not possible to include them all. Both N6-methyladenosine (m6A) and adenosine-to-inosine (A-to-I) editing are the most abundant RNA modifications at adenosines. Generally speaking, the catalytic mechanism of the two RNA modifications is different. Although processed with distinct catalytic mechanism, an intriguing question is whether m6A and A-to-I are always regulated independently. Both the presence and extent of A-to-I sites in m6A-negative RNA transcripts suggest there is a negative correlation between m6A and A-to-I [97].

## Translational potential of AZINs for anticancer therapy

AZIN1 has been a regulator of the polyamine synthesis pathway, and also has roles independent of polyamines, which play crucial roles in regulation of cell growth and carcinogenesis. Emerging data of polyamine influence on tumor microenvironment indicate that antitumor mechanisms of polyamine-targeting therapies extend beyond direct manipulation of polyamine levels in cancer cells. RNA editing level of AZIN1 is higher in many cancer types than that in corresponding non-cancerous tissues. Hyperediting of AZIN1 has turned out to be prognostic factor for survival and metastasis in many cancer types. Therefore, identification of suppressing AZIN1 editing may contribute to the development of novel RNA therapeutics and increase success rate of cancer treatment.

### A potential target for anticancer drug

As previously described, AZIN1 has been shown to regulate various biological processes not limited to the polyamine pathway. Then, developing AZIN1 inhibitor for cancer therapy may be superior to targeting polyamine synthetic pathway for the reason that inhibiting intracellular synthesis could lead to polyamine uptake from extracellular fluid by means of compensatory mechanism. Besides, targeting AZIN1 can inhibit cell proliferation through blocking polyamine pathway and promoting degradation of various oncoproteins regulated by AZIN1. Moreover, we have found that RNA-edited AZIN1 could also change the sensitivity to some anti-cancer drugs, such as decreasing the sensitivity of transfected Ba/F3 cells to IGF-1R inhibitor [25]. Therefore, AZIN1 may be an emerging target for anticancer drug development.

### Prognosis predictor

First, the integrative copy-number aberration and gene expression analyses indicated that AZIN1 gene expression increased in a focal amplification of 8q22.3 and associated with metastatic recurrence [78], which identified AZIN1 as a novel driver of metastatic progression in high-risk prostate cancer. Then, elevated editing frequency of AZIN1 has also emerged as a prognostic factor for overall survival and disease-free survival and an independent risk factor for lymph node and distant metastasis of CRC [87]. Next, high edited AZIN1 could also be a predictor of worse prognosis in endometrial cancer [98] and prostate cancer [95]. These findings of AZIN1 as the prognosis predictor strongly suggest that suppressing AZIN1 editing may contribute to the development of novel RNA therapies for cancer treatment in multiple cancer types.

In contrast, AZIN2 expression is a signature of EMT-associated secretory phenotype that is linked to an adverse prognosis in CRC [99]. High AZIN2 expression was related to well-differentiated histological type with a functioning vesicle transportation system in adenoid cystic carcinoma (ACC) tissue, which suggested that AZIN2 could be a prognostic factor for better survival of ACC patients [100].

### Drug discovery

The potential efficacy of targeting AZIN1-OAZ1 interactions using small molecules, peptides, or proteins as a therapeutic strategy for cancer treatment is viable upon rigorous validation [101, 102]. There are two possible ways to support the above assertion. Firstly, AZIN1 exerts precise control over the polyamine biosynthetic pathway, thereby regulating cellular proliferation through the sequestration of OAZ1. Although the inhibition of ODC using DFMO [103], a well-known ODC inhibitor, effectively suppresses intracellular polyamine synthesis, it does not impede the uptake of polyamines from tumor microenvironments. Conversely, the inhibition of AZIN-OAZ1 interaction may potentially release OAZ1, leading to the degradation of ODC and a subsequent reduction in polyamine uptake. Additionally, AZIN1 plays a crucial role in regulating cell cycle progression by preventing OAZ1-mediated degradation of proteins associated with cell-cycle regulation. Consequently, inhibiting the interaction between AZIN1 and OAZ1 may potentially hinder AZIN1-mediated cell proliferation.

**Fig. 5:**
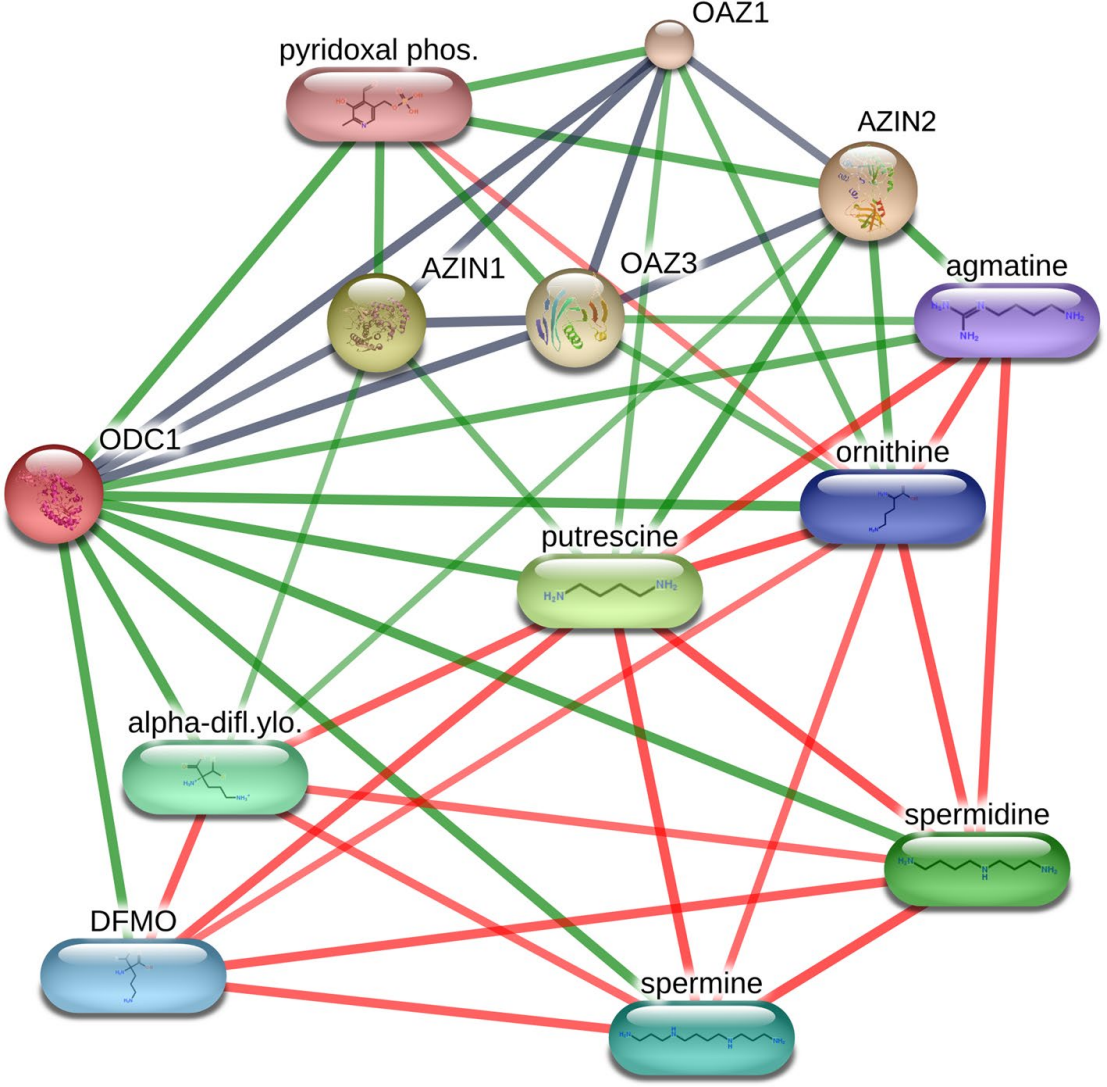
The protein–protein and protein-drug interaction network of AZIN1 and AZIN2. The interaction network with high confidence levels (> 0.7) is shown as displayed by The European Molecular Biology Laboratory's Search Tool for the chemical association networks database (STITCH) version 5.0 for identifying protein-drug interaction. Stereo-isomers are shown as separate compounds. Line thickness indicates the strength of data support. Red line indicates the presence of fusion evidence. Green line indicates neighborhood evidence. Black line indicates coexpression evidence. alpha-difl.ylo: alpha-difluoromethylornithine; pyridoxal phos: pyridoxal phosphate

To identify the roles of AZI /analogs family, we used AZIN1, AZIN2, and ODC as entry item to explore protein–drug interactions through analysis of STITCH database (The European Molecular Biology Laboratory's Search Tool for the chemical association networks database), containing known and predicted interactions of chemicals and proteins [104]. Interaction with confidence level exceeds 0.7 can be kept. It shows that AZIN1 has a strong connection with those proteins (OAZ1 and OAZ3) related to regulating polyamine metabolism. Interestingly, the drug-protein interaction network identified that alpha-difluoromethylornithine and pyridoxal phosphate are tightly correlated with AZIN1, which have not been mentioned in previous reports (Fig. 5). This analysis revealed that metabolism‐related regulatory proteins related with AZIN1 may be potential targets for anticancer drug. A biomarker study on cancer can be undermined if the suggested biomarkers are found in other cancers, which compromises their specificity. A more specific and effective AZIN1 inhibitor is required, however, for therapeutic intervention in a wide range of cancers. Although RNA therapeutics targeting cancer associated RNA editing have not yet been introduced into the clinic, the chemically modified antisense oligonucleotides (ASOs)-mediated inhibition of AZIN1 editing effectively suppresses tumor incidence and growth, suggesting that many cancer patients with high editing level of AZIN1, may benefit from the AZIN1-targeting and ASO-based therapeutics [105], which is being developed as a novel and promising class of drug.

## Concluding remarks

AZINs play a crucial role in growth and development of organisms. Indeed, there is no doubt that dysregulation of AZINs has involved in multiple human malignant diseases, notably precancerous lesions and cancers. AZINs regulate tumor growth, migration and invasion through polyamine and other pathways. Besides, we review RNA editing, as one of RNA modification types on AZIN1 in human diseases, and A-to-I RNA editing of AZIN1 also has involved in cancer progression. The upregulation of RNA-edited AZIN1 in human cancers correlates with increased tumor progression and aggressiveness. RNA-edited AZIN1 enhances CRC stemness, promotes tumor angiogenesis, and appears to drive the metastatic processes. However, knowledge is limited about differences in mechanisms between wild and edited-type AZIN1 in cancers so far. As is illustrated in liver cancer, A-to-I RNA editing of AZIN1 may facilitate tumorigenesis through neutralizing antizyme mediated degradation of ODC and cyclin D. Furthermore, the nuclear translocation of RNA-edited AZIN1 is required for increased tumor aggressiveness.

Herein, current research revealed that AZIN1 RNA editing and its role in tumorigenesis could be a potential prognosis predictor and therapy target. Notably, emerging data demonstrating influence of AZIN1 on cancer stemness and microenvironment indicate that anticancer strategy should not be limited to direct manipulation of AZIN1 in cancer cells. Although, RNA therapeutics targeting cancer-driven or associated RNA editing events have not yet been identified or introduced into the clinic, AZIN1 may serve as a therapeutic target for anticancer drug development superior to polyamine synthesis inhibitors and warrants further studies.

## Data Availability

Not applicable.
